# Security Analysis for Smart Healthcare Systems

**DOI:** 10.3390/s24113375

**Published:** 2024-05-24

**Authors:** Mariam Ibrahim, Abdallah Al-Wadi, Ruba Elhafiz

**Affiliations:** Department of Mechatronics Engineering, German Jordanian University, Amman 11180, Jordan; a.alwadi1@gju.edu.jo (A.A.-W.); r.elhafiz@gju.edu.jo (R.E.)

**Keywords:** Internet of Medical Things (IoMT), good health and well-being, Intrusion Detection System (IDS), Machine Learning (ML), Deep Learning (DL), Honeypot, Artificial Intelligence (AI), ensemble method

## Abstract

The healthcare industry went through reformation by integrating the Internet of Medical Things (IoMT) to enable data harnessing by transmission mediums from different devices, about patients to healthcare staff devices, for further analysis through cloud-based servers for proper diagnosis of patients, yielding efficient and accurate results. However, IoMT technology is accompanied by a set of drawbacks in terms of security risks and vulnerabilities, such as violating and exposing patients’ sensitive and confidential data. Further, the network traffic data is prone to interception attacks caused by a wireless type of communication and alteration of data, which could cause unwanted outcomes. The advocated scheme provides insight into a robust Intrusion Detection System (IDS) for IoMT networks. It leverages a honeypot to divert attackers away from critical systems, reducing the attack surface. Additionally, the IDS employs an ensemble method combining Logistic Regression and K-Nearest Neighbor algorithms. This approach harnesses the strengths of both algorithms to improve attack detection accuracy and robustness. This work analyzes the impact, performance, accuracy, and precision outcomes of the used model on two IoMT-related datasets which contain multiple attack types such as Man-In-The-Middle (MITM), Data Injection, and Distributed Denial of Services (DDOS). The yielded results showed that the proposed ensemble method was effective in detecting intrusion attempts and classifying them as attacks or normal network traffic, with a high accuracy of 92.5% for the first dataset and 99.54% for the second dataset and a precision of 96.74% for the first dataset and 99.228% for the second dataset.

## 1. Introduction

The technological advancement led to the augmenting of electronics with bio-interaction capabilities that interact on a machine-to-human level, which enhanced health conditions in terms of preserving and sustaining the well-being of humans [1] by leveraging the available technologies that can record and monitor vital readings remotely and contentiously [2] without the continuous presence and availability of the medical staff, as well as using past recorded medical data and analytics for tracking early indications of medical conditions that may arise. Through cloud-sharing services, the functionality of IoMT in the medical field has improved by harnessing and utilizing the computing and processing performance of electronics that are built and designed for caring and performing diagnostics when treating and monitoring patients [3]. An application of IoMT is the usage of sensors that collect patient health data through implantable devices, either internally or via wearables that can be worn around different parts of the body. Data such as heart rate, oxygen saturation, and insulin levels are routed and sent to physicians’ monitoring devices, which are connected to local servers or remote cloud-based data banks for further analysis. Utilizing modern examination instruments and advanced tools that are reliant on electronics and connections, whether hardwired or wireless, will yield better protection and preventative care to combat current and future diseases based on experience and collected data [4].

For the viable merging and integration of IoT into the health sector, the medical records had to be digitized, which made the use of IoMT possible, besides the utilization of Artificial Intelligence (AI), to assist the medical staff with their daily care for patients. IoMT integration is accompanied by risks and challenges associated with the cybersecurity field in terms of securing connections and handling data transmission [5]. Analyzing the networking terminology, applications, communications methods, and concerns related to securing such systems is considered crucial to mitigate these risks. A list of security concerns has been identified in terms of data integrity, management, risk assessment, private data, threats, and vulnerabilities. The IoMT network creates a huge load of data, which requires a lot of computing power and resources to handle and deal with it. The remote transmission of data through wireless mediums will create vulnerabilities and opportunities for cybercriminals to utilize and take advantage of, compromising the integrity and confidentiality of healthcare data and causing devastating harm.

Multiple methods are used to secure the information, including cryptography, blockchain, quantum computing, and two-factor authentication for handling and ensuring the integrity of Wireless Body Area Networks (WBAN) data confidentiality [6]. Biometrics and digital signatures are used to verify the authenticity and strengthen the security measures of transmission, which ensure integrity and prevent eavesdropping or interception as it is hard to decipher and tamper with the data. Hashing is also used to encrypt the data, as it converts the passwords into fixed-size strings of characters, a process that is irreversible as it is a one-way process [7]. In terms of access control, the data is secured by implementing defensive and secure mechanisms such as role-based access control, whose functionality is to restrict access to resources based on the user’s functional roles and responsibilities by revoking or enforcing the privilege-based access level provided. Multifactor authentication, such as smart cards and biometrics, requires a one-time code for accessing the required data. Firewalls are implemented to monitor the inbound and outbound traffic between trusted internal networks and external, unknown networks, preventing unauthorized access and malicious attacks. Virtual Private Networks (VPNs) are used to encode the data transmission for secured remote access when required over communication channels. Vulnerabilities are also present in cognitive radio systems and are a security concern, requiring access control methods of authentication and authorization to ensure the integrity of the data. Quantum cryptography for radio systems and their vulnerabilities has a modulation method to encrypt the message using a noise loop modulation for the physical layer security [8]. Intrusion detection and prevention methods come into play when safeguarding the information and data, as they are capable of detecting and responding to activities and potential breaches in real time, analyzing the network, identifying abnormal patterns for automated actions to be taken, or blocking or mitigating threats.

Challenges are presented when trying to secure the IoMT with methods that render them ineffective due to limited storage capacity and slow computational ability when processing the collected data. The integration of AI tools such as Machine Learning (ML) and Deep Learning (DL) methods into IoMT systems is motivated by the necessity to analyze and investigate their effects on boosting and enhancing the stability, security, and scalability of the health system. Generating large amounts of data could be handled and computed through the use of appropriate classification techniques, in addition to incorporating and integrating them with an Intrusion Detection System (IDS) that utilizes both the power capabilities of AI and modern methods to enhance the integrity and security of healthcare systems [9]. The novelty of the proposed work lies in developing an AI-powered IDS framework that utilizes a honeypot to categorize and investigate cyber threats in Smart Healthcare Systems (SHSs). This requires the selected model to be simulated with various datasets that deal with IDS and IoMT. An in-depth simulation analysis and performance parameters of this model will be presented in this paper.

The usage of an ensemble learning model combining Logistic Regression (LR) and K-Nearest-Neighbor (KNN) for Intrusion Detection Systems (IDSs) integrated with honeypot and tested out with IoMT datasets can be justified for several reasons [10]. Firstly, the IDS usually encounters multiple and diverse types of attack types that vary in their characteristics and complexities. LR and KNN offer complementary strengths in handling different types of data and detecting anomalies and patterns while analyzing the data. LR strength lies in the capability of detecting linear types of data and effectively functions on the probability of an event occurring based on the input features. The KNN is a non-parametric algorithm as it can learn any functional form from the training dataset and is capable of detecting and capturing complex non-linear relationships between features and the case of abnormal occurrences. The combination of LR and KNN in an ensemble model utilizes both of the algorithms’ functionality, enhancing the IDS system’s capability of detecting a broad-spectrum range of attacks as they tend to be robust and resilient to overfitting, due to aggregating predictions from multiple base learners that provide reduced risk of bias and variance. The ensemble method output produces enhanced detection accuracy when integrated with the honeypot, as well as increased robustness to evolving threats, as incorporating the honeypot has improved the detection rate and boosted the early warning system in addition to being scalable and flexible for adaptation. The integrated system of the IDS honeypot ensemble model also provides continuously evolving data about the attacker behavior pattern that is observed by the change in the emulated network inside the honeypot, which also increases the accuracy and overall performance of the system [11], making it more effective in identifying and mitigating security threats in real time.

The proposed framework architecture aims to provide an enhanced and secure machine learning model capable of intrusion detection and prevention mechanisms. The key contributions and objectives include:

- Ensuring the integrity of medical data within the IoMT architecture by proposing an enhanced architecture and secure mechanism for data transmission.
- Validating the results through two IoMT-related datasets by integrating them with an ensemble-powered IDS honeypot threat detection and mitigating system, ranging from biometrics collected from patients to network flow and attack patterns.
- Developing an integrated intrusion detection and prevention system with machine learning that runs an ensemble method combining Logistic Regression and K-Nearest-Neighbor for an architecture that is scalable and deployable for IoMT network structures and is integrated with honeypot.

## 2. Related Work

Various IDS techniques have been investigated in the literature; for instance, an IDS technique using Blockchain (BC) technology is proposed in [12] that runs the Federated Learning method, a machine learning technique that trains an algorithm via multiple independent sessions, with each using its dataset and trained locally by the model, hence preserving the privacy of data. To prevent and avoid attacks on healthcare systems, the proposed model uses the Dwarf Mongoose Artificial Neural Network (DMO-ANN) optimization model, which operates with limited memory usage and network boundaries at high performance and can deal with a lot of data generated by IoT devices in healthcare. Using a cloud IDS-based architecture for data loss prevention, in addition to placing the model along the network’s periphery near the attacker access point, yields enhanced speed in the detection of intrusion attempts.

Another model that utilizes and combines multiple methods is the Best Estimator, Random Forest, and GridSearchCV algorithms [13]. The WUSTL-EHMS-2020 dataset was generated by employing a real-time Enhanced Healthcare Monitoring System (EHMS) test bed. The dataset contains a combination of biometrics that were collected from both network flow metrics and patient biometrics. Dimension reduction was implemented due to benign features for accelerated determination of the output. The types of cyberattacks captured and included in this dataset are limited only to man-in-the-middle attacks: data injection and spoofing. The IDS is responsible for capturing real-time network traffic abnormalities and the patient’s biometric data. After capturing the packets from the network traffic via the Audit Record Generation and Utilization System (ARGUS) [14] tool, the dataset produced normal traffic labeled as 0 and attack traffic as 1.

An anomaly detection method was addressed in [15] by proposing an ensemble approach for unsupervised anomaly detection in honeypot data by building upon an existing algorithm such as the Local Outlier Factor (LOF). The improvements were implemented by enhancing unsupervised anomaly detection within honeypot data by modifying the subset of data features and adjusting the parameters of the anomaly detection algorithm. The applied outlier ensemble method tackles the diversity of the anomalies by searching for abnormal patterns in various data subspaces and implementing the outlier detection algorithm with various parameters. The general approach of the proposed Parameterization and Feature Subspace-based Outlier Ensemble (PFSOE) algorithm was applied at Kyoto University, with a dataset consisting of daily captured traffic network data from 2006 to 2015.

The authors’ suggested approach in [16] was to address the challenge and issue of traditional static honeypots, which are susceptible to detection by anti-honeypot technologies, which cause a significant reduction in the effectiveness of honeypots by attracting and observing real attacker patterns. The proposed method functions by having a dynamically distributed honeypot system based on blockchain, whereby the honeypots are distributed across the network, making them more difficult for attackers to identify. Additionally, the system mechanism utilizes blockchain technology to store honeypot deployment information and access logs, ensuring data integrity and immutability. The key features of this method are that it makes the network defenses better at hindering intruders’ reconnaissance and improving deception effectiveness. Integrating blockchain technology for storing and managing information about honeypot deployments and access logs enhances security and transparency within the system.

A Hybrid Deep Learning (HDL)-based classification model was employed in [17], which integrates the Convolutional Neural Network (CNN) and Long Short-Term Memory (LSTM) models for intrusion detection systems. This method enables secure transmission of IoT devices in the healthcare industry to detect intrusion attempts while transmitting medical data. The Flower Pollination Algorithm (FPA) is used to optimize hyperparameters and the process of tuning the HDL, yielding an enhanced detection rate. The experimental outcome of the model was tested on the ToN-IoT [18] and CICIDS-2017 [19] datasets, respectively. The cross-validation of hyper-parameter optimization of the HDL model and FPA helps boost the predictive outcome of the model for unseen data. Latency issues might arise due to a lack of optimizer algorithms.

A Multi-class classification Intrusion Detection Model (M-IDM) for healthcare IoT is proposed in [20] by employing a Convolutional Neural Network (CNN). It operates by seeking the byte sequence of the network traffic and known hostile malware patterns. The dataset was generated from real devices from the National Cancer Center in South Korea, and it was segregated into multiple classification features, such as minor, major, informal, and critical, for both signature-based and anomaly-based patterns, regarding their impact on the network traffic. The classification of intrusion procedures was separated into a Network Intrusion Detection System (NIDS) and a Host-based Intrusion Detection System (HIDS). The analysis of network traffic was employed by NIDS for better performance and prediction of anomaly detection. The HIDS sends alerts in cases of suspected activity while monitoring critical operating system files.

The authors of [21] used a Fog-Based Attack Detection (FBAD) framework by utilizing an Ensemble of Online Sequential Extreme Learning Machines (EOS-ELM) that detects malicious activities. Due to its deployment at the network’s periphery near the IoT devices, the technique displayed high accuracy and lower latency. The performance of the framework based on the architecture demonstrated better results compared to other methods in terms of having a distributed architecture outperforming a centralized type and yielding detection and classification accuracy rates that cause a reduction of limitations on cloud-based services due to employing the fog layer on the architecture.

An ensemble approach was proposed by the authors in [22] for IoT networks to be implemented by the IDS to identify and analyze the network for any malicious patterns. The objective is to enhance the IDS’s effectiveness and performance in detecting attacks and classifying network traffic as normal and abnormal. They employed two ensemble approaches, which included voting and stacking mechanisms, which were used to improve the classification accuracy of the output model. They employed multiple supervised machine learning algorithms: K-Nearest-Neighbor, Decision Tree, Logistic Regression, and Random Forest. The stacking model performed better compared to the voting model and performed well in terms of accuracy, precision, and recall.

## 3. Architecture of IoMT

**Figure 1:**
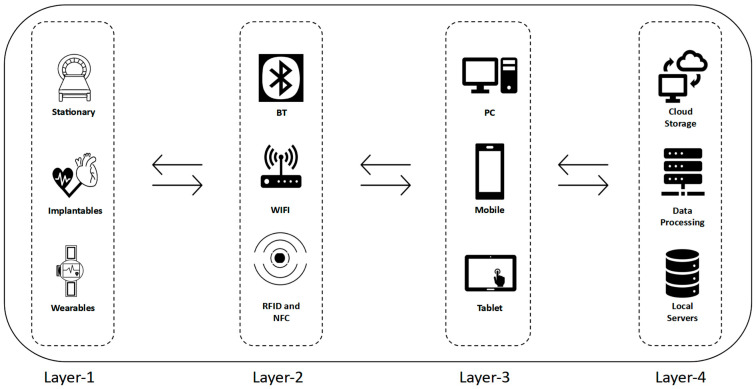
IoMT System Architecture.

The IoMT architecture depicted in Figure 1 is focused on the interaction between human-to-object, medical assets, and equipment ranging from wearable, stationary, and implantable, which transmit and transfer medical data via multiple methods for the analysis and diagnosis of medical conditions.

The structure of IoMT consists of four main layers, with each responsible for specific main functions and operations as follows:

- Layer 1: Devices and Instruments Layer, which deploys sensors and actuators to obtain and collect patients’ data. Three main variants are categorized according to their applications and functionality: stationary, wearable, and implantable.
  1. Medical examination instruments that are stationary, such as CT scans, MRI machines, and X-rays for imaging purposes [23]. Additionally, some of the surgical operations are performed with high-speed and low-latency connections [24] via a remote controller for operating robotic surgical instruments and apparatus, which outperform some operations done by hand in terms of precision and accuracy [25]. Medications and prescriptions that are accurately prescribed with the right dosage are capable of adapting based on the diagnosed medical condition and history of the patient and are dispensed accordingly [26].
  2. Surgically implanted devices inside the human body, or semi-invasive devices. An example would be a pacemaker, which regulates heart rate and rhythm by sending electrical pulses, or an insulin pump, which delivers insulin into the body through a computerized device with regulated doses at a specific preset time [27].
  3. Wearable sensors and noninvasive medical equipment transmit medical data through wired or wireless mediums to monitoring and analytical devices that are operated by the healthcare staff [28]. Wristbands, smartwatches, and Oxylink devices transmit medical reading data to be analyzed for detecting and determining abnormalities and harvesting collected data for research and studies. Such readings measure the heart rate, oxygen saturation, and blood pressure [29].
- Layer 2: Communication Network Layer, a bridging layer responsible for transmitting IoMT data from Layer 1 to Layer 3. Wireless transmission variant methods [30] are applicable based on the requirements of the device or type of data, such as short-range communication, which includes Radio Frequency Identification (RFID), used for logistics and inventory management, Near-Field Communication (NFC), such as tags that contain the Identification (ID) credentials of patients, Bluetooth (BT) devices, such as Oxylink, and Wireless Fidelity (WiFi) for remote patient monitoring [31].
- Layer 3: Application interface monitoring layer. The purpose of this layer is the aggregation and collection of different medical device readings and the conversion of the collected medical data into specific format standards that facilitate the interoperability and interaction of medical devices [32]. The main functions are detecting, predicting, tracking, and recording the patient data. Establishing a common standard would allow communication between different systems and devices to handle the data and implement encryption and authentication privileges for access to safeguard the confidentiality and sensitive data of patients. This layer also facilitates real-time monitoring of all medical equipment readings for any abrupt or slight occurrences of abnormalities and utilizes a friendly Graphical User Interface (GUI) design [33] for ease of use and access to live data that can be read and understood. Securing this layer is crucial, as there is a reliance on it for accessing medical records remotely from cloud servers or local servers from Layer 4 with smooth communication and a low-latency network. The network structure and architecture of the IoMT should establish and maintain secure and strict connection protocols for accessing information, which can be ensured by securing the servers and gateways and employing different encryption and defensive mechanisms.
- Layer 4: Data processing and storage layer, storing the data on local servers as well as utilizing remote services such as cloud servers for performing high computational analysis of collected medical data as it is more cost-effective and efficient compared to using local servers in terms of maintenance, security, redundancy, and scalability in the long run [34]. This layer serves as the backbone of the IoMT main system architecture, as there is a high demand for intensive computational requirements and big data analytics in the medical field, which would require an augmentation with AI technology to provide the best care and treatment procedures by accessing past medical histories of patients which have been harvested and collected in large amounts and shared across different cloud platforms [35]. Due to the access to cloud services, it is possible to apply parallel computing to provide the accelerated best course of action scenarios during diagnostics of cases, surgeries done manually or by robotics, synthesizing new drugs, and research and development [36].

## 4. IDS Architecture

**Figure 2:**
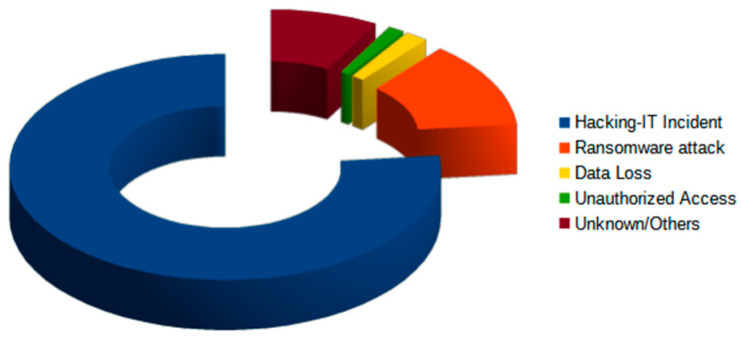
Cyberattack effects on individuals.

**Figure 3:**
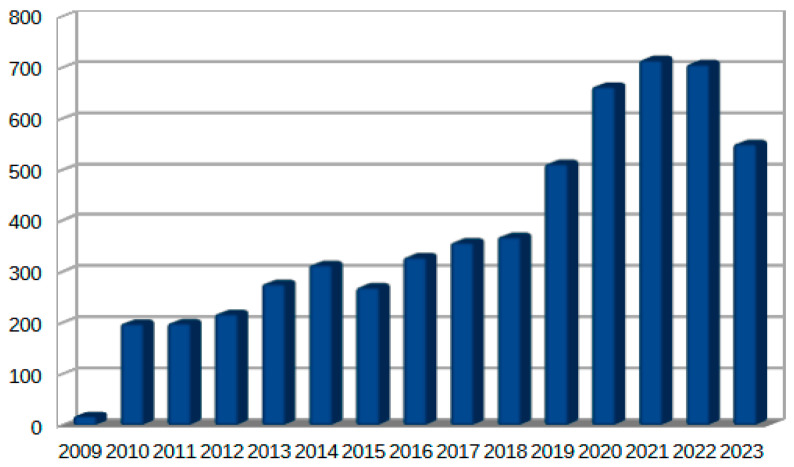
Cyberattack occurrences.

The integration of interconnected cyber-physical devices in the health sector requires an intensive harvesting process that generates a huge amount of data aggregated and processed in IoMT systems. This resulted in many cyberattacks and caused damage to the healthcare infrastructure, as well as damage on an individual level, as many people were affected. Integrity breaches of security were gathered and documented, as shown in Figure 2 and Figure 3, respectively, depicting the incidents that occurred from 2009 until 2023 as reported by the Department of Health and Human Services, Office for Civil Rights (OCR) in the U.S. [37]. An amount of 287,709,238 affected individuals were recorded, emphasizing the necessity of an adaptable security system that is agile and capable of securing the network from any emerging threats.

Healthcare cyberattacks affect multiple aspects in terms of the financial and organizational, as well as on personal levels, and the main reason for most of the crippling effects is hacking-IT incidents. In terms of financial aspects, this results in devastating capital losses due to fraud and ransoms. Organizational impact, in terms of losing the image as a safe institute that safeguards patients’ private data and losing trust in the long run, leads to a reduction in productivity, as the IT operations are disrupted by ransomware and data leaks. On a personal level, many individuals suffer from identity theft and financial loss [38].

Due to the necessity and requirements of securing medical data transfer these days, IoMT system infrastructures are required to be designed to be effective in safeguarding the integrity of the collected sensitive and confidential data of healthcare workers and patients. The functionality of an IDS tool is to provide preemptive mitigation for detecting and preventing cyberattacks by identifying the risks and threats that could disrupt and compromise the IoMT’s functionality and performance [39]. An IDS is an essential requirement in any IoT-based system built and designed for monitoring the network traffic between connected devices to detect irregular activities and classify them as anomalies inside the network. The IDS is internally installed in the IoMT network to increase its detection range and efficiency for alerting the security administrators of any potential breaches and attempts at unauthorized access. The IDS tool actively seeks these abnormalities and detects them based on multiple factors and detection methods, such as:

- Signature-based type of detection, which requires predefined attack patterns and known exploits that are previously recorded and regularly updated through cloud security firms, aggregating the collected cyberattack occurrences from multiple IoT sectors and implementing them into the current IDS mechanism for better performance in detection [40].
- Anomaly-based type, which requires an in-depth examination of network interactions by integrating AI technology into the IDS, such as machine and deep learning algorithms for monitoring the system network activities by distinguishing normal network behavior and any deviations and anomalies that are in contrast to real-time generated network traffic, user activities, and system configurations. Anomaly-based systems are effective in the detection of zero-day attacks with no predefined signatures, as they rely on disturbances and abrupt normal patterns [41].

An IDS performance could be further enhanced by tackling certain challenges and fine-tuning some properties in terms of:

- Producing false positive feedback alerts due to failing to detect an anomaly of a cyberattack that is sophisticated in nature, as well as stealthy attacks. Continuously fine-tuning and validating the records of such alerts would scale up to improve the accuracy and precision of detection, thereby minimizing false alarms, which is achievable by utilizing AI methods [42].
- An IDS mechanism must be capable of adapting and scaling up with an expanding network structure while maintaining optimal performance. An IDS that is high-performing by design is capable of adjusting to new network traffic, a higher-density network, and an increased flow of data that is aggregated and transported. The IDS must also be designed to integrate with existing mechanisms for providing additional risk management and rapid response [43].

**Figure 4:**
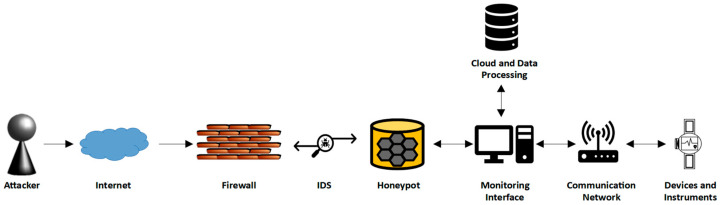
IDS-integrated IoMT Network Architecture.

The proposed framework architecture actively detects and mitigates threats as it contains multiple components in terms of security tools that may keep the network safe, such as a firewall [44], an AI-based IDS, and a specific type of IDS called a honeypot [45]. The honeypot mechanism relies on deception and diversion methods for detection and prevention to deflect and reroute the attackers by luring and entrapping them inside a heterogeneous environment that mimics the real network traffic [46]. In addition to providing existing vulnerabilities and exploits inside the simulated environment that entice the attacker by weakened systems to latch on and enable the monitoring and studying of the behavior of the malicious actors by capturing their activities for intelligence gathering and fore-sighting future risks and newly developed cyberattack patterns [47]. This will assist the research and development institutes in creating countermeasures to mitigate the threats without risking the main network environment of the IoMT system’s critical assets. Figure 4 provides a diagram of the suggested IDS technique.

The interaction levels of honeypots vary depending on their operation, from lower to higher levels based on their resource intensity requirements, as there are virtual and physical honeypots [48]. A higher interaction level is required due to the critical aspect of safeguarding the IoMT system components, as they are crucial infrastructure. The higher level is resource-intensive to maintain with higher operational costs and solid infrastructure, which is justified due to the robust detection and interaction capabilities in providing more in-depth detailed patterns and advanced analysis in addition to understanding the attacker techniques [49].

The honeypot provides real services that are capable of replicating the operating systems and applications of a real-time network environment and their functionality in terms of logging activities and IoMT device interactions in-depth analysis, which have proactive defense in terms of automated responses in alerting and monitoring any suspicious activities and breach attempts [50]. The scalability of the honeypot is in two orientations in terms of horizontal scaling, which involves deploying multiple honeypots to increase coverage of network traffic, and vertical scaling, which involves increasing the capability of individual honeypots for in-depth analysis [51]. Among the essential elements of the honeypot is the isolation of the central IoMT network, ensuring the inability of intrusive attempts and malicious actors to impact and cause disturbances [52].

The historical datasets are collected for preprocessing stages and validations to be fed into AI algorithms for further study and in-depth analysis with better accuracy. A large and dense amount of data can be sorted and inspected by utilizing an AI method that specifically deals with classification, as the IDS will sort out the collected data and classify it as an anomaly or normal traffic. Due to the limitations of publicly available datasets that are specifically generated for the IoMT environment, only two datasets were found to be applicable for the evaluation of the model framework:

- Dataset 1: The WUSTL dataset [53] is from a test bed that was created by using the real-time Enhanced Healthcare Monitoring System (EHMS). It is used to train an IDS AI-based model to detect certain attacks that are recorded and tabulated in the dataset. The type of attack is mainly Man-In-The-Middle (MITM) [54], which consists of spoofing [55] and data injection attacks [56]. It contains flow network metrics in addition to patient biometrics. This dataset consists of 44 features, and the attacker patterns are labeled as 1, while the normal patterns are labeled as 0.
- Dataset 2: The IoT-Flock tool [57] was used to generate an IoT traffic network that consists of a normal and attack traffic network for malicious activity detection in healthcare environment setup [58]. Normal traffic contains patient and environment monitoring devices under normal conditions. The attacker traffic was generated after the normal traffic with multiple types of attacks, including a DDOS-type attack MQTT [59], a MQTT publish flood [60], brute force [61], and a SlowITE attack [62]. The dataset contains 52 features, with the attacks labeled as 1 in the attack dataset and normal traffic labeled as 0 in the dataset. Table 1 provides details about the datasets.

The selection of two datasets is merely to showcase the impact of different aspects of the model parameter output and its performance and effectiveness. Both datasets are medical IoT-related to the type of attacks, and the network traffic recorded volume differs greatly in terms of quantity. The building and development of the ML algorithm require preprocessing of the selected datasets, the selection of features, training, and testing the ML model to validate the results. Data preprocessing [63] is mandatory and essential in machine learning optimization, ensuring that the data are appropriately formatted, cleaned, and transformed to fit the model and improve its performance and reliability [64]. Here are the common preprocessing stages for datasets In machine learning:

- Both datasets were created by capturing the network traffic in their respective environments as pcap files, which are network characteristics that will be used for analysis. For these files to be analyzed, they must be converted into CSV files, which is done by using a Python 3.12 script.
- Cleaning the data of irrelevant values in terms of handling and removing missing values, noise, errors, and any inconsistency. Data transformation is required for the classification algorithm to scale the features to a numerical range via binary encoding and ensure the consistency of selected features. Merging and combining multiple datasets of normal and attack samples to create a unified, comprehensive dataset for analysis.
- Normalization of data ensures that all features have similar scales, which assists the model in converging quicker during the training phase and prevents certain features from affecting others by dominating them.
- Data reduction is implemented to improve the model efficiency by feature selection and identifying the relevant data that impact the outcome of the model by removing low variances among the features as they have less information. This is done via an iterative process such as recursive feature elimination, which begins with all features and removes the least impactful ones by using the Support Vector Machine (SVM) algorithm. The features selected to train and test the machine learning model for the two datasets are:
  - Dataset 1: [‘SrcLoad’,‘DstLoad’,‘SrcJitter’,‘DstJitter’,‘Dur’,‘Load’,‘Rate’]
  - Dataset 2: [‘frame.time_delta’,‘tcp.time_delta’,‘tcp.flags.ack’,‘tcp.flags.push’, ‘tcp.flags.reset’,‘mqtt.hdrflags’,‘mqtt.msgtype’,‘mqtt.qos’,‘mqtt.retain’,‘mqtt.ver’]

Choosing relevant features is critical for a model’s performance, so the selection process underwent careful consideration.

After the data is preprocessed, the next stage is training and testing, which involves splitting the dataset randomly with a ratio of 75% allocated for training and the remaining 25% for testing. Afterward, the model is trained to detect network traffic anomalies in the IoMT environment. Lastly, the model is tested to evaluate its performance in anomaly detection in IoMT network traffic.

**Figure 5:**
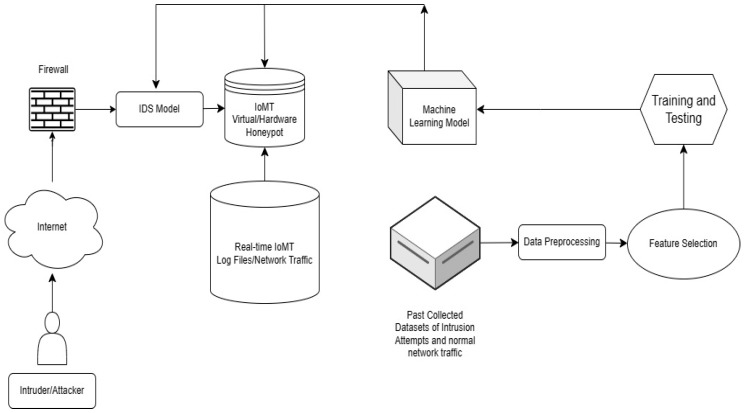
Proposed Framework Architecture.

The proposed framework architecture in Figure 5 is capable of detecting and mitigating threats due to having access to previously collected datasets of intrusion attempts and normal network traffic that are collected from cloud-sharing sources of cybersecurity institutes. This enables the IDS and honeypot to be in a continuous state of patching process with up-to-date data on newly discovered signature-based threats. The IDS is tuned for detection, and the honeypot is enhanced to emulate normal and attack patterns in a network, as it is necessary for enticing the attackers and deceiving them by validating their action and showing a response from the honeypot that they are achieving their target while their actions are being researched and analyzed. These collected data also go through preprocessing stages before training the machine learning model. The honeypot environment is connected to the real-time IoMT network traffic environment to provide enticing bait for the attacker to latch on, which enables the study of the attacker’s behavioral patterns. Algorithm 1 shows the pseudocode for the Integrated IDS and Honeypot with Ensemble Method using LR and KNN.
**Algorithm 1**: Integrated IDS and Honeypot with Ensemble Method using LR and KNNInput
oAccept a dataset containing network traffic records, labeled as ‘normal’ or ‘intrusion’.IDS Initialization
oSplit the dataset into training and testing sets.oInitialize Logistic Regression (LR) and K-Nearest Neighbor (KNN) models.Model Training
oFor each record in the training set, do the following:
▪Train the LR model.▪Train the KNN model.Model Testing
oFor each record in the testing set, do the following:
▪Make a prediction using the LR model.▪Make a prediction using the KNN model.▪Use ensemble learning to combine the predictions.▪Compare the ensemble prediction with the actual label to evaluate the performance.Honeypot Initialization
oSet up a system to emulate network traffic.IDS Monitoring and Honeypot Deception
oProcess initialization:
▪Preprocess the data to extract features.▪Use the trained LR and KNN models to predict (‘normal’ or ‘intrusion’).▪If the prediction is ‘intrusion’:
Alert the system administrator.Redirect the traffic to the honeypot.The honeypot emulates the cyberattack behavioral pattern to deceive the intruder.
▪Otherwise:
Allow the traffic to continue normally.
Output
oIntrusion detection alerts.oClassified traffic (normal/attack).

A scenario where it is assumed that the intruder bypassed the firewall defensive mechanism and gained access to the network by utilizing multiple methods could be because:

- Misconfigurations found in the firewall protocols create loopholes that grant the intruder access inside the network via overly permissive rules or incorrect settings, which are exploited by the attacker [65].
- Vulnerabilities in firewall rules are exploited by employing sophisticated attacks that target the applications or services via zero-day [66] attacks, as the firewall is not regularly maintained and updated and is incapable of blocking unknown threats and vulnerabilities.

After bypassing the firewall, the intruder is lured into the honeypot, unaware of being monitored by the IDS. The proposed framework architecture functionality is based on intruder activities, which are categorized as follows:

- Using a signature-based approach, the IDS will cross-reference the activity of the network, compare it with historical datasets, and classify it as an attack. It then notifies the security personnel of this intrusion attempt and flags it as it enters the honeypot-emulated environment for further study of the intruder activity. The honeypot mechanism purposely mimics existing vulnerabilities for the intruder to latch on by actively monitoring the logs and activities in the simulated environment.
- Using an anomaly-based attack that deviates from the normal behavioral pattern of the network traffic and known cyberattacks. The AI-based IDS continuously monitors the activity of the network and keeps track of the normal patterns. If any abnormalities and disturbances in the honeypot occur, they get classified as anomalies by comparing them and cross-validating them with normal network behavior.

The proposed framework architecture utilizes an ensemble voting classifier machine learning model [67], which combines multiple base classifiers to justify the final selected prediction. The ensemble method is used to enhance the overall performance of IDS by utilizing Logistic Regression (LR) [68] and K-Nearest Neighbor (KNN) [69] algorithms, as each model compensates for the limitations of the other model and increases the effectiveness of the outcome results. The justification for choosing both algorithms is based on the following properties:

- LR advantages for IDS can be driven by its interpretability, as it can provide a clear understanding of the result of each feature on the prediction, especially when the data variables are approximately linear. In terms of resource management, it is computationally efficient for large datasets or real-time applications, fast and simple, as it can be adapted for online usage, and the model is updated incrementally as new data becomes available. Considerations and drawbacks of LR underperformance include the assumption that the relationship between the features is linear.
- The KNN algorithm is capable of capturing complex non-linear relationships in the dataset due to its distribution-free method of statistical analysis. Flexibility and adaptability to different types of data distributions do not require a specific decision boundary. KNN does not require an explicit training phase, which makes it versatile for implementation and continuous data updates. KNN is resource-intensive compared to LR, as it involves computing all the training samples and has high sensitivity when there is feature scaling.

A comprehensive review of multiple ID methods for the IoMT environment is shown in Table 2.

All of the data goes through multiple stages, from data preprocessing to feature selection to training and testing, before implementing the machine learning algorithms, which are tailored to the specific needs based on the datasets provided to enhance the accuracy and predictive functionality of the IDS and update its database for any slight or drastic changes that deviate from the prerecorded attacks. Implementation and the integration of AI technology significantly improved the application and defensive mechanisms of the IoMT cyber sector. An AI-driven environment will increase the probability of safeguarding the data. In this proposed framework and based on the available datasets, security measures were put in place to provide a versatile and safe environment for healthcare data. The machine learning classifiers are evaluated based on several metrics that assess their performance in making predictions on new, unseen data [73]. This includes accuracy, True Positive Rate (TPR), False Positive Rate (FPR), specificity, recall, and F1-score. The following four fundamental values form the basis of these standards:

- True Positive (TP): The number of normal samples that have been correctly classified as normal.
- True Negative (TN): The number of attack samples that have been correctly classified as an attack.
- False Positive (FP): The number of attack samples that have been classified as normal.
- False Negative (FN): The number of normal samples that have been detected as an attack.

Accuracy represents the percentage of correctly classified attacks of the total packets and normal traffic network:

(1) $$Accuracy=\frac{TP+TN}{TP+FN+TN+FP}\times 100\%$$

Precision is the number of true positive predictions divided by the sum of true positives and false positives. Precision focuses on the accuracy of positive predictions:

(2) $$Precision=\frac{TP}{TP+FP}\times 100\%$$

Recall (also called True Positive Rate, TPR) is the number of true positive predictions divided by the sum of true positives and false negatives. Recall focuses on the ability of the classifier to capture all positive instances:

(3) $$Recall\left(TPR\right)=\frac{TP}{TP+FN}\times 100\%$$

F1-Score is the harmonic mean of precision and recall. It provides the balance between precision and recall:

(4) $$F1-Score=2\times \frac{Precision\times Recall}{Precision+Recall}\%$$

The FPR is defined as the ratio between the wrong attack detection and all the normal samples:

(5) $$FalsePositiveRate\left(FPR\right)=\frac{FP}{FP+TN}\times 100\%$$

The Area Under the Curve (AUC) Receiver Operating Characteristics (ROC) curve demonstrates the true positive rate and false positive rate trade-off.

The Area Under the Curve (AUC) Precision-Recall (PR) curve measures the area under the precision-recall curve and is beneficial for imbalanced datasets.

## 5. Results and Discussion

**Figure 6:**
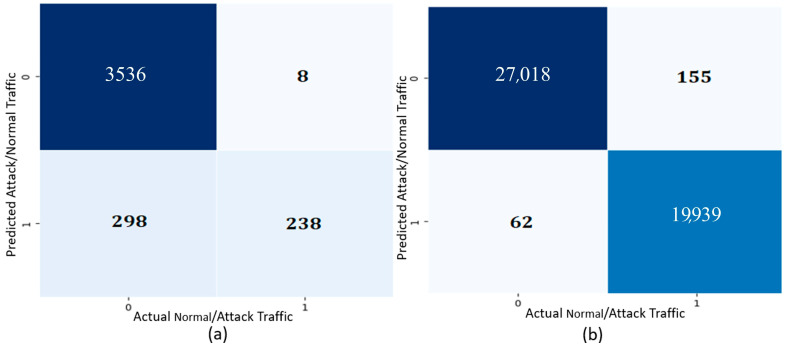
(**a**) Dataset 1 Confusion Matrix and (**b**) Dataset 2 Confusion Matrix.

The ensemble method utilized for the IDS was evaluated by multiple parameters and executed by Jupyter Notebook, which is a web-based program for coding purposes. The adaptation of machine learning models with IDS output parameters is provided in Table 3. Figure 6a,b depicts the confusion matrices of the ensemble method for the two datasets.

The value of 298 in the confusion matrix of Dataset 1 shows that the model is capable of classifying normal network traffic, but because of the absence of attack samples compared to normal samples in the dataset (as indicated in Table 1 earlier), there is an imbalance of samples, which causes the model to be biased towards the normal class.

The performance parameters of Dataset 2 show that the model is classifying normal and attack network traffic at a high rate, which yields fewer false alarms and more accurate results as compared to Dataset 1.

**Figure 7:**
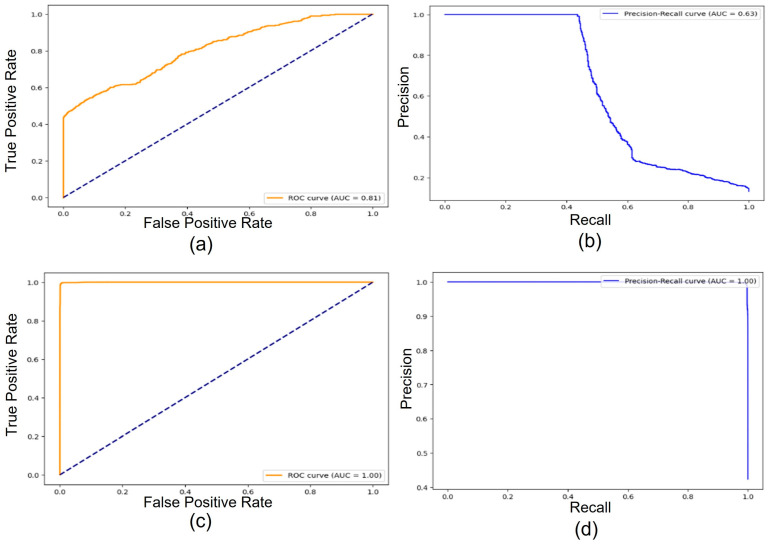
(**a**) Dataset-1 AUC-ROC graph, (**b**) Dataset-1 AUC-PR graph, (**c**) Dataset-2 AUC-ROC graph, and (**d**) Dataset-2 AUC-PR graph.

Figure 7a–d shows the AUC-ROC and AUC-PR for the two datasets, respectively [74]. These curves are crucial metrics for evaluating binary classification models. However, they assess performance in different ways, making them suitable for various scenarios.

**AUC-ROC**

- Focus: Measures the model’s ability to distinguish between positive and negative classes at various classification thresholds.
- Curve: Plots the True Positive Rate (TPR) on the *y*-axis against the False Positive Rate (FPR) on the *x*-axis.
  - TPR (Recall): Proportion of positive cases correctly identified.
  - FPR (Fall-out): Proportion of negative cases incorrectly classified as positive.
- Interpretation:
  - A perfect model has an AUC-ROC of 1, indicating a perfect separation between classes at all thresholds.
  - An AUC-ROC of 0.5 represents random guessing.
  - Higher AUC-ROC signifies a better ability to differentiate classes across thresholds.

**AUC-PR**

- Focus: Measures the model’s ability to rank positive cases correctly, considering both precision and recall.
- Curve: Plots the precision (proportion of predicted positives that are actually positive) on the *y*-axis against the recall on the *x*-axis.
- Interpretation:
  - A perfect model has an AUC-PR of 1, indicating that all predicted positives are true positives and the model ranks them correctly.
  - An AUC-PR of 0.5 suggests no better than the random ranking of positive cases.
  - Higher AUC-PR signifies better ability to identify true positives and prioritize them appropriately.

Several factors can influence the AUC values:

- Classification Threshold: Changing the decision threshold for classifying positive cases will affect both TPR and FPR, consequently impacting AUC-ROC.
- Data Distribution: The underlying distribution of positive and negative classes can influence the difficulty of separation and thus the AUC values.
- Model Complexity: Overly complex models might lead to overfitting, reducing generalization, and potentially lowering AUC on unseen data.
- Class Imbalance: As mentioned earlier, class imbalance can significantly affect AUC-ROC, but might have less impact on AUC-PR.

AUC-ROC and AUC-PR evaluate the model’s performance in distinguishing and ranking positive cases. It can be noticed that the model in Dataset 2, being balanced, showed better class separation and true positive identification as compared to the imbalanced model in Dataset 1.

The experiments demonstrated how the trained AI-based ensemble method provides better results due to the combination of multiple models that yielded an accuracy of 92.5% compared to the work done on the previously mentioned WUSTL dataset [53]. In their model, they showed a test accuracy score comparison of different singular models, which produced 92.13%, 92%, 92.4%, and 90.04 for the Random Forest (RF), K-Nearest-Neighbor, Support Vector Machine, and Artificial Neural Network, respectively. Using an ensemble method produces slightly better results in terms of comparison and is better suited for future analytics implemented on datasets, as future datasets may have diverse types of data in them in terms of being linear or nonlinear to the output, which the ensemble model can adapt to and produce enhanced results for, compared to singular models. The IDS model of the proposed framework can detect cyberattacks to secure and protect the IoMT network environment by using the ensemble method for better performance parameters. The strategic deployment of the honeypot enhances the security measures, as the mechanism provides early indications of changes in the network, and by integrating it with an ensemble method it is even enhanced compared to standard honeypots, as they are only capable of detecting signature-based changes while integrating it with ensemble, provided the functionality of detecting anomaly-based types of attacks. Depending on the dataset features and the number of normal network traffic and attacks, the parameters of the model were affected. The variance of the resulting output parameters in terms of F1-Score and Recall was impacted in Dataset-1 due to the imbalance of normal and attack samples in the dataset, unlike what was observed in Dataset-2.

## 6. Conclusions

The increase in dependency on IoMT has raised many security concerns about safeguarding the health network environment as cyberattacks pose a potentially crippling effect on the network structure. In this paper, the proposed framework architecture for the IoMT utilizes an AI-based Intrusion Detection System (IDS) integrated with a honeypot to increase the defensive mechanism of the IoMT network environment and safeguard confidential data. The machine learning models that were used are Logistic Regression and K-Nearest Neighbor, which were utilized in an ensemble method for better parameter performance. The experiments were trained and conducted on two publicly available IoMT-related datasets that contain normal and attack samples. The results showed that the proposed ensemble method was effective at detecting intrusion attempts and classifying them as attacks or normal network traffic with a high accuracy of 92.5% for the first dataset and 99.54% for the second dataset and a precision of 96.74% for the first dataset and 99.228% for the second dataset, in addition to other parameters, while maintaining optimal performance. The design considerations are resource-efficient in terms of resource management, computational complexity, resource management, and scalability, in addition to the adaptability of handling large datasets. Regarding future work, it would be difficult to determine the performance parameters due to the limitation of accessing real-time services in the IoMT realistic environment, which provides live continuous feedback inside the network but has a high probability of capturing more accurate results. It would also require the utilization of more datasets containing more variants of cyberattacks, since the IoMT-related datasets are scarce or inaccessible due to regulations, as the generated datasets in a simulated environment produce different results compared to the IoMT-realistic environment. Future work could be implemented to further boost the functionality and increase the defensive mechanism against cyberattacks when it is in continuous feedback mode, which is achieved by integrating multiple sources of data. Such sources are real-time IoMT network traffic, the emulated environment of the honeypot, and a cloud-shared historical database of known attack patterns. The yielded scenario of combining the three sources can go through a continuous cross-validation process to detect anomalies and be more effective at detecting zero-day-type attacks. Once an attack is classified as an intrusion, it is later registered and added to the historical dataset for future reference; then, it gets shared via different platforms and cloud-sharing services across different cybersecurity institutes. Due to the limitation of accessing real-time traffic network data in an IoMT-related environment, it is currently not possible to determine the performance parameters of such an architecture.

## Figures and Tables

**Table 1:** Datasets details.

Measurement	Dataset Size	Number of Normal Samples	Number of Attack Samples	Total Number of Samples
Dataset 1	4.4 MB	14,272 (87.5%)	2046 (12.5%)	16,318
Dataset 2	102 MB	108,568 (57.54%)	80,126 (42.46%)	188,694

**Table 2:** Comparison of ID methods.

Feature	Signature-Based IDS [70]	Honeypot [71]	Machine Learning-Based IDS [72]	Integrated Machine Learning and Signature-Based IDS with Honeypot (Proposed Model)
Detection Method	Relies on pre-defined signatures to match known attack patterns.	Lures attackers into a decoy system mimicking real network services to observe their behavior.	Learns from network traffic data to identify patterns indicative of attacks.	Combines machine learning for pattern recognition with signature-based detection for known threats. Honeypot lures attackers to gather further intel.
Strength	- Fast and efficient. - Lower false positives (with well-established signatures). - Explainable decisions based on matched signatures.	- Detects zero-day attacks and Advanced Persistent Threats (APTs). Provides rich attacker behavior data for analysis. - Can be used for attacker profiling and deception.	- Adaptable to novel attacks. - Continuous learning improves detection accuracy. - Can automatically identify relevant features.	- Adaptable to novel attacks with machine learning. - Faster detection with a signature-based approach. - Rich attacker behavior data from the honeypot - Provides insights into attacker techniques and tools.
Considerations	- Limited adaptability to unseen attacks. - Requires constant signature updates to stay effective. - Evasion techniques can bypass signature-based detection. - Generally simpler to deploy and manage.	- Requires careful configuration to mimic real systems effectively. - Limited scalability for large deployments. - Potential security risks if compromised. - Requires careful configuration and isolation to avoid compromising real systems. Expertise in honeypot analysis is essential.	- Computationally expensive (training and running models). - Susceptible to false positives due to model biases or data limitations. - Black box nature: decision-making process might be less interpretable. - Generally, more complex, requiring expertise for setup, configuration, and maintenance.	- Increased complexity in deployment and maintenance. - Requires expertise in both machine learning and honeypot analysis. - Potential for false positives due to model biases or data limitations.

**Table 3:** Performance Parameters.

Parameters	Dataset-1	Dataset-2
Accuracy	92.5	99.54
Precision	96.74	99.228
Recall	44.402	99.69
F1-Score	60.869	99.458

## Data Availability

The original contributions presented in the study are included in the article, further inquiries can be directed to the corresponding author.
